# Children neuropsychological and behavioral scale-revision 2016 in the early detection of autism spectrum disorder

**DOI:** 10.3389/fpsyt.2022.893226

**Published:** 2022-07-22

**Authors:** Shuling Chen, Jinzhu Zhao, Xiaolin Hu, Lina Tang, Jinhui Li, Dandan Wu, Tian Yan, Lu Xu, Min Chen, Shan Huang, Yan Hao

**Affiliations:** ^1^Division of Child Healthcare, Department of Pediatrics, Tongji Hospital, Tongji Medical College, Huazhong University of Science and Technology, Wuhan, China; ^2^Department of Child Health Care, Wuhan Children’s Hospital, Tongji Medical College, Huazhong University of Science and Technology, Wuhan, China

**Keywords:** Children Neuropsychological and Behavioral Scale-Revision 2016, communication warning behavior, autism spectrum disorders, screen, early detection

## Abstract

**Background:**

The Children Neuropsychological and Behavioral Scale-Revision 2016 (CNBS-R2016) is a widely used developmental assessment tool for children aged 0–6 years in China. The communication warning behavior subscale of CNBS-R2016 is used to assess the symptoms of autism spectrum disorder (ASD), and its value of >30 points indicates ASD based on CNBS-R2016. However, we observed that children with relatively lower values were also diagnosed with ASD later on in clinical practice. Thus, this study aimed to identify the suitable cutoff value for ASD screening recommended by the communication warning behavior of CNBS-R2016.

**Materials and methods:**

A total of 90 typically developing (TD) children and 316 children with developmental disorders such as ASD, developmental language disorder (DLD), and global developmental delay (GDD; 130 in the ASD group, 100 in the DLD group, and 86 in the GDD group) were enrolled in this study. All subjects were evaluated based on the CNBS-R2016. The newly recommended cutoff value of communication warning behavior for screening ASD was analyzed with receiver operating curves.

**Results:**

Children in the ASD group presented with lower developmental levels than TD, DLD, and GDD groups in overall developmental quotient assessed by CNBS-R2016. We compared the consistency between the scores of communication warning behavior subscale and Autism Behavior Checklist (ABC), Childhood Autism Rating Scale (CARS), Autism Diagnostic Observation Schedule, second edition (ADOS-2), and clinical diagnosis for the classification of ASD at a value of 30 based on the previously and newly recommended cutoff value of 12 by the CNBS-R2016. The *Kappa* values between the communication warning behavior and ABC, CARS, ADOS-2, and clinical diagnosis were 0.494, 0.476, 0.137, and 0.529, respectively, with an agreement rate of 76.90%, 76.26%, 52.03%, and 82.27%, respectively, when the cutoff point was 30. The corresponding *Kappa* values were 0.891, 0.816, 0.613, and 0.844, respectively, and the corresponding agreement rate was 94.62%, 90.82%, 90.54%, and 93.10%, respectively, when the cutoff point was 12.

**Conclusion:**

The communication warning behavior subscale of CNBS-R2016 is important for screening ASD. When the communication warning behavior score is 12 points or greater, considerable attention and further comprehensive diagnostic evaluation for ASD are required to achieve the early detection and diagnosis of ASD in children.

## Introduction

Autism spectrum disorder (ASD) is a life-long neurodevelopmental disorder characterized by persistent impairments of social communication and restricted and repetitive behavior (1), with incidences rapidly increasing worldwide. According to Centers for Disease Control and Prevention of American, the prevalence of ASD is as high as 1/54 in children before the age of 8 (2). The latest national survey shows that the prevalence of ASD in children aged 6–12 years in China is 0.70% (95% CI: 0.64–0.74%) (3), which is generally lower than that in the United States, indicating the possibility of many unidentified cases.

Autism spectrum disorder is a serious disease that affects children’s social adaptability. A national sample survey has shown that ASD is the leading cause of disability among disabled children aged 6 or younger in China (4). ASD not only affects children’s families, but also causes a huge economic burden to the society (5, 6). Studies have shown that early behavioral treatment can largely improve the cognitive and adaptive abilities of children with ASD (7, 8). In general, the earlier the intervention, the better the outcomes (9–12). Early screening and early diagnosis play a key role in the prognosis of this disease (13, 14). Signs of ASD can occur very early, and the symptoms could usually be captured before the age of 2 (14–17), such as the lack of social smile at the age of 6 months, the lack of orientation to his or her name at the age of 12 months, and inability to point at things at the age of 15 months (18–21). However, at present, the diagnosis of ASD is performed around the age of 4–5 years on average (22, 23). A delay can be observed between the onset of ASD symptoms and diagnosis. Therefore, research about early screening, particularly the screening tools, can continue to effectively optimize and accelerate diagnostic procedures.

Some imported tools, such as the Checklist for Autism in Toddlers, Modified Checklist for Autism in Toddlers, Autism Behavior Checklist (ABC), and Clancy Autism Behavior Checklist, have been used for screening ASD (24, 25). Although these tools have been commonly applied in municipal maternity and children’s healthcare/(tertiary) hospitals and primary medical institutions in some large cities in China, these scales are rarely utilized in community health service centers and district maternity and child healthcare hospitals (24) as well as difficult to use as routine well-child visit items because of culture and cost factors (26–28). Moreover, the existing screening tools in clinical practice are suitable for a limited age group, whereas the age range of target assessment objects varies greatly, and the screening stages used are not the same. Some scale copyrights are more restricted. Therefore, at present, effective screening tools in Chinese are lacking.

The Children Neuropsychological and Behavioral Scale is an indigenous development assessment tool with Chinese norms that was developed by the Capital Institute of Pediatrics of China (29) since the early 1980s. Researchers designed the test items in accordance with developmental rules and behavioral characteristics of Chinese infants. These items were verified and completed in a cross-sectional study of 1,275 children aged 0–4 years and further standardized in 15,053 children from 12 representative provinces and cities through strict nationwide sampling. Finally, 177 items were included in the children neuropsychological and behavioral scale for young ones aged 0–4 years. The five subscales, namely, gross motor, personal social, language, fine motor, and adaptive behavior, were consistent with the relevant subscales in Gesell (30) and demonstrated adequate reliability in children with typical development. The scale was revised from 2005 to 2016 to include new items and expand the age range and standardized sample size and then named the Children Neuropsychological and Behavioral Scale-Revision 2016 (CNBS-R2016). The CNBS-R2016 includes 294 items, and the test age was expanded to 6 years. A new subscale called communication warning behavior was added apart from the five subscales to assess the symptoms of ASD. Communication warning behavior contains 33 items, including social communication disorder, restricted and repetitive behavior, language, sensory abnormalities, physiological disorder, intelligence, and abnormal behavior. CNBS-R2016 is a widely used developmental assessment scale at various levels of medical institutions in China, particularly in maternal and child healthcare and primary care hospitals, as a part of routine well-child visits.

Some of developmental behavioral pediatricians or child healthcare physicians in China have screened and detected ASD based on the communication warning behavior. A point of the communication warning behavior over than 30 points indicates ASD as suggested by CNBS-R2016. However, we observed that children with lower values of communication warning behavior were also diagnosed with ASD later on in clinical practice. Therefore, we aimed to study the suitable cutoff value for screening ASD and provide suggestions about the application of CNBS-R2016 in the early detection of ASD in children.

## Materials and methods

### Participants

Children aged 2–5 years who visited the outpatient Division of Child Healthcare, Department of Pediatrics, Tongji Hospital, Tongji Medical College, Huazhong University of Science and Technology from March 2019 to December 2021 were enrolled in this study. Children with developmental disorders and typically developing (TD) children were recruited. Developmental disorders included ASD, developmental language disorder (DLD), and global developmental delay (GDD). All participants completed developmental assessment of the CNBS-R2016. ASD and GDD groups were diagnosed based on the ASD criteria of American Psychiatric Association Diagnostic and Statistical Manual of Mental Disorders 5th edition (DSM-V) (31) and further confirmed by the Autism Diagnostic Observation Schedule, second edition (ADOS-2). GDD refers to children aged under 5 years with profound delay of ≥2 standard deviations below the mean in two or more developmental domains (32), and children who showed abnormal results in gross motor and fine motor domains without any other backward domain were excluded in this study. The DLD group was diagnosed based on the diagnostic criteria of the ICD-11 (33) and backward only in language. Children in the TD group were those without any developmental disorders and with normal results of CNBS-R2016 and who were recruited in the same period from routine well-child visits. The parents or caregivers of the children who agreed to participate in this study were provided with informed consent.

### Instruments

#### CNBS-R2016

All children included in this study participated in the developmental assessment by the CNBS-R2016. The mean value of the general developmental quotient (DQ) and the five subscale quotients of the CNBS-R2016 is 100. A subscale quotient of less than 70 points (<2 standard deviations [SDs]) indicates a developmental delay; a quotient between 70 and 79 points is slightly below the threshold for developmental delay, and a quotient greater than or equal to 80 points showed no developmental delay (29). The communication warning behavior subscale of CNBS-R2016 has a total of 33 items, including the core characteristics of ASD such as reducing social interaction ability; inappropriate communication styles; repetitive behavior; the lack of shared attention, sympathy, and imagination; and physiological disorder in infant. It is assessed through questioning or interactive observation. Items that affect social interaction function are assigned with higher values, which are similar to the Autism Behavior Checklist. Based on the original opinion, a score less than 7 points shows less possibility of ASD; a score between 7 and 12 points indicates a need for follow-up; a score between 12 and 30 points indicates a risk of communication and interaction disorder, and a score greater than 30 points indicates a high possibility of ASD.

#### ABC and childhood autism rating scale

The ABC is a behavior questionnaire that is completed by child’s parents or caregivers. The questionnaire covering five aspects of autism symptoms: sensory, relating, body concept and object use, language, social and self-care. Items are scored on a 4-point scale, ranging from 0 (no problem) to 3 (severe problem). The higher the score, the more serious the problem (34). The standard cutoff value was 53, and a score above 53 points indicated high probability of ASD (35). The Childhood Autism Rating Scale (CARS) is a clinician-completed tool to rate the presence and severity of ASD by incorporating information from caregivers’ reports and direct observation. A score of ≥30 points indicates a possible diagnosis of ASD (36, 37). The ABC and CARS are commonly used scales in clinical practice and ASD research. The higher the scores on the two scales, the more severe the autism symptoms.

#### Autism diagnostic observation scale-2

The Autism Diagnostic Observation Scale-2 (ADOS-2) is a standardized and partly structured tool that provides a standardized assessment of ASD symptoms. It is a play-based, semi-structured assessment tool used to assess communication, social interaction, and restricted and repetitive behavior in individuals with ASD, which forms the part of the recommended “gold standard” for the diagnosis of ASD (38). ADOS-2 can be used as a diagnostic assessment for children aged 12 months and above with possible ASD. It includes five modules (module T and module 1–4), and the selection of different modules depends on the age and language expression level. Based on DSM-V, ADOS-2 is composed of two parts: social affect (SA) and repetitive behavior (RRB) (39). The total score (TA) is the combined score of these two parts by following a specific algorithm. In eliminating the effect of age, TA will be transferred to the corresponding calibrated severity (CSS) score in accordance with a standardized conversion table provided by ADOS-2. The CSS of each module has a cutoff point corresponding to the diagnostic criteria. The higher scores of ADOS-2 CSS, the more severe of the autism symptoms.

### Procedure

We introduced this project to 685 parents and children, 434 of which agreed to participate in this project, including 341 children with developmental disorders and 90 typically developing children. Of the 341 children with developmental disorder, 25 were excluded because of incomplete medical records, and they did not meet the inclusion criteria.

These subjects were recruited in accordance with a standardized process. During the first visit to the hospital, children suspected of developmental delay received an initial inquiry approximately 20 min by an outpatient developmental behavioral pediatrician. This process collected information about children’s current health condition, developmental status, and family history. For children highly suspected of ASD, the outpatient pediatrician would schedule the evaluation, including ABC, CARS, CNBS-R2016, and ADOS-2. The parents completed the ABC by following the instruction of another developmental pediatrician; meanwhile, this pediatrician completed the CARS by observing children’s behavior and interviewing their parents or guardians. A trained and qualified developmental pediatrician would complete the CNBS-R2016 during children’s first visit. The ADOS-2 was scheduled within 1 week by a certified developmental pediatrician. It was completed in an assessment room approximately 20 m^2^ in size, and approximately 1 h was needed for each child. DLD and GDD were recruited simultaneously in accordance with the corresponding diagnostic criteria. All DLD and GDD children recruited in this study were assessed by CNBS-R2016, ABC, and CARS. Some diagnoses could not be distinguished from ASD, and ADOS-2 was scheduled as necessary. The later scale evaluators were blinded to the initial diagnosis of outpatient pediatrician to avoid bias. The hearing and vision of all children with developmental disorders were examined to exclude disabilities caused by serious hearing and vision loss. Brain MRI, EEG, molecular genetics, and metabolic test were scheduled optionally. TD children were recruited as the control group. They did not have any developmental problems, and they were assessed in accordance with the CNBS-R2016 as a part of the routine physical examination.

**FIGURE 1:**
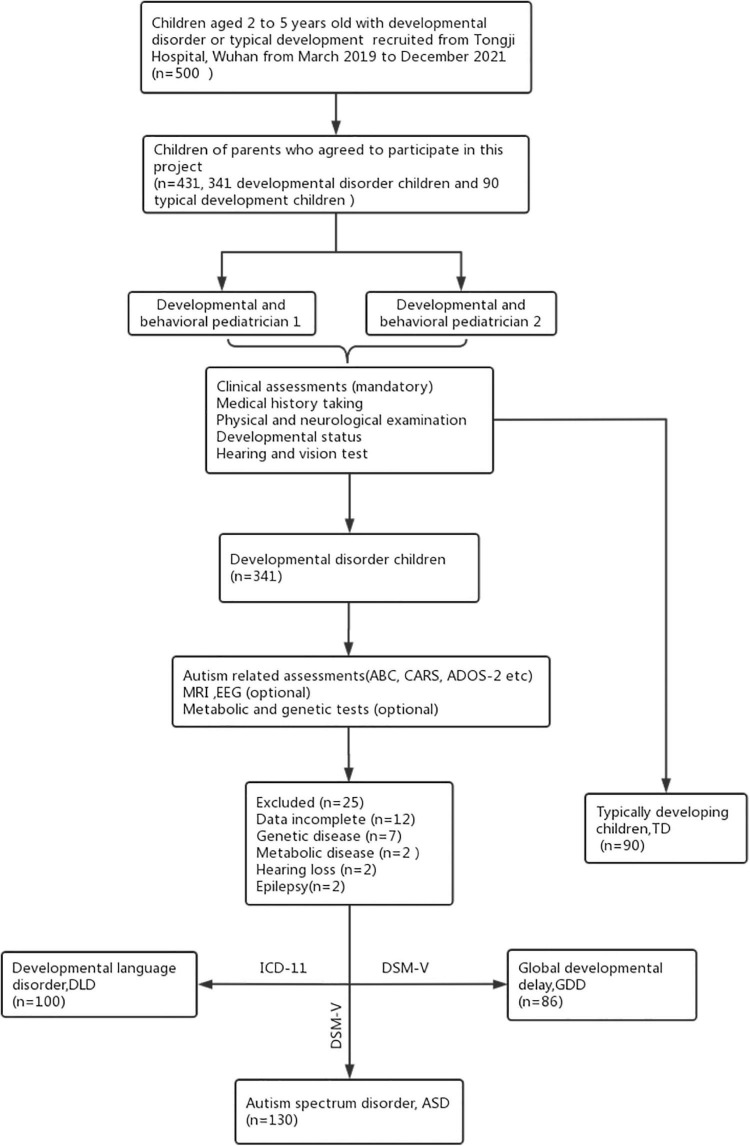
Recruitment and diagnosis of participants.

The final diagnoses were established by integrating data from parent interviews, developmental status, medical records, information provided by other caregivers and teachers, and direct observation and interaction with children during at least two assessment visits. All diagnoses were confirmed by two developmental and behavioral pediatricians. Participants were excluded if they had any diseases of the nervous system, deafness, selective mutism, or other difficulties with known biomedical conditions such as metabolic or genetic diagnoses, and so on. The recruitment and diagnosis of participants are shown in Figure 1.

### Data analysis

Data analyses were performed using SPSS 22.0 and GraphPad Prism 6.0. The Kolmogorov–Smirnov test was used to determine the distribution of the analyzed variable before analysis. Continuous normal variables are described as means ± SDs. Non-normal distribution of variables is described as median (P25 and P75). Categorical variables are described as frequencies and percentages. One-way analysis of variance (ANOVA) was performed to compare age, subscale, and DQ scores among all groups (TD, ASD, DLD, and GDD), and the least significant difference (LSD) *post hoc* test was performed for multiple comparison. In addition, the Kruskal–Wails *H* test was conducted to compare communication warning behavior scores of four groups. The chi-squared test (χ^2^) was used for categorical variables. Spearman’s correlations were conducted to examine the relationship between the communication warning behavior scores and established autism scales (ABC, CARS, and ADOS-2). *P* < 0.05 was considered statistically significant.

The ability of the communication warning behavior to predict the diagnostic category for each of the cutoffs was examined by receiver operating characteristic (ROC) analysis. In evaluating the accuracy of the diagnostic instrument, the area under the curve (AUC) was used. Based on the criteria of Swets et al. (40), the AUC value was interpreted as low diagnostic accuracy for AUC < 0.7, moderate diagnostic accuracy for AUC ranging from 0.7 to 0.9, and high diagnostic accuracy for AUC > 0.9. For each optimal cutoff point, the false-negative rate (FNR; proportion of true positive that is mistook as negative), false-positive rate (FPR; proportion of true negative that is mistook as positive), positive predictive value (PPV; proportion of a positive test result that is true positive), and negative predictive value (NPV; proportion of a negative test result that is true negative) were calculated. The consistency among the communication warning behavior of CNBS-R2016 and ASD screening tools (ABC and CARS), ASD diagnostic tool ADOS-2, and clinical diagnosis for the classification of ASD was expressed as *Kappa* value and agreement rate (AR; proportion of true and negative results). *Kappa* ≤ 0.2, *Kappa* ranging from 0.4–0.6, and *Kappa* ≥ 0.6 were interpreted as poor, moderate, and excellent consistency, respectively (41). With regard to ABC, children with a score ≥ 68 points were classified as ASD. With regard to CARS, children with a score ≥ 30 points were classified as ASD. As for ADOS-2, children who were given moderate or severe attention based on module T and who obtained ASD diagnosis based on modules 1–4 were deemed as positive results and classified as ASD.

## Results

### Characteristics of the study population

A total of four hundred and six cases aged 2–5 years were included in final analyses. Among them, 130 children fulfilled the diagnostic criteria of ASD, 100 children of DLD, 86 children of GDD, and 90 TD children. Table 1 presents the demographic characteristics and developmental levels of participants assessed by CNBS-R2016 in each of these four groups. No significant differences in gender and age were observed among these four groups. With regard to developmental levels assessed by CNBS-R2016, four groups differed significantly with one another in six subscales of DQs and overall DQs (Table 1, *p* < 0.05).

**TABLE 1:** Demographics and developmental levels of participants.

	ASD (a)	DLD (b)	GDD (c)	TD (d)	*X^2^/F/H*	Overall group comparison *P*	*Post hoc* comparisons
	(*n* = 130)	(*n* = 100)	(*n* = 86)	(*n* = 90)			
Male (n%)	104 (80.00)	78 (78.00)	69 (80.23)	69 (76.67)	0.501*	>0.05	
Age (year)	3.09 ± 0.73	3.01 ± 0.66	3.08 ± 0.65	3.17 ± 0.66	0.936**	0.423	
Gross motor	77.30 ± 15.64	93.31 ± 9.89	67.27 ± 10.54	104.87 ± 9.15	177.827**	<0.001	c <a < b < d
Fine motor	54.02 ± 14.55	78.14 ± 10.29	60.43 ± 10.32	96.36 ± 10.73	258.113**	<0.001	a <c < b < d
Adaptive behavior	61.62 ± 17.73	86.75 ± 11.66	67.63 ± 10.22	110.52 ± 12.65	252.655**	<0.001	a <c < b < d
Language	40.08 ± 15.48	47.98 ± 9.86	56.15 ± 9.26	106.42 ± 12.32	570.711**	<0.001	a <b < c < d
Personal-social	54.56 ± 13.13	75.25 ± 8.99	61.03 ± 8.24	107.96 ± 11.99	458.398**	<0.001	a <c < b < d
Developmental quotient	57.52 ± 12.49	76.36 ± 5.96	62.20 ± 5.43	105.21 ± 5.69	625.364**	<0.001	a <c < b < d
Communication warning behavior	24 (16, 32.25)	2 (0, 4)	8 (4, 13)	0 (0, 0)	303.251***	<0.001	d <b < c < a

TD (a), typically developing children; ASD (b), autism spectrum disorder; DLD (c), developmental language disorder; GDD (d), global developmental delay. Data of communication warning behavior were shown in the format of median (P25, P75). *Data analyzed with chi-squared test. **Data analyzed with one-way ANOVA test. ***Data analyzed with Kruskal–Wails H test. The overall group comparison serves as an omnibus test comparing the means, medians, or ratio between four groups. Post hoc comparisons with step-up LSD correction. Two-sided at significance level of 0.05.

### Distribution of communication warning behavior score in different groups

**FIGURE 2:**
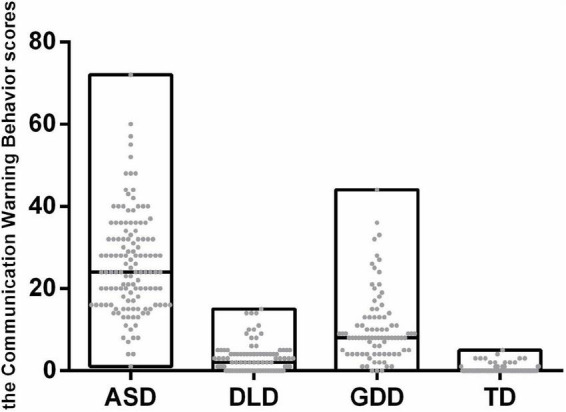
Communication warning behavior score distribution of different groups.

The distribution of communication warning behavior scores of all four groups is shown in Figure 2. The communication warning behavior scores in CNBS-R2016 in children with ASD, DLD, GDD, and TD ranged from 1 to 72, 0 to 15, 0 to 44, and 0 to 5, respectively, and the median score of four groups was 24, 8, 2, and 0, respectively. The ASD group scored dramatically higher than the other three groups (Table 1, *p* < 0.05).

The correlation between quotients of communication warning behavior and established autism scales in ASD children is presented in Table 2. Correlation coefficients between communication warning behavior scores and ABC, CARS, and ADOS-2 CSS were 0.812, 0.761, and 0.821, respectively, which were all positive and significant (*p* < 0.05).

**TABLE 2:** Correlations between the communication warning behavior scores and established autism scales (*n* = 130).

	ABC	CARS	ADOS-2 CSS
Mean ± SD	67.16 ± 26.46	33.73 ± 8.40	17.97 ± 4.64
*r*	0.812	0.761	0.821
*p*	<0.05	<0.05	<0.05

ABC, Autism Behavior Checklist; CARS, Childhood Autism Rating Scale; ADOS-2 CSS, calibrated severity score of ADOS-2. P < 0.05 stands for significant correlation.

The communication warning behavior of CNBS-R2016 score of >30 points was selected for the prediction of ASD, and consistency with ABC, CARS, ADOS-2, and clinical diagnosis is shown in Table 3. Consistency among them was moderate except for ADOS-2, which was limited by a number of children who completed an ADOS-2 assessment. However, almost all children with 30 points and above were classified within ASD, and only three children were diagnosed as non-ASD.

**TABLE 3:** Consistency between the communication warning behavior (cutoff value = 30) of CNBS-R2016 and ABC, CARS, ADOS-2, and clinical diagnosis for the classification of ASD.

		ABC		CARS		ADOS-2		Clinical diagnosis	
		ASD	Non-ASD	ASD	Non-ASD	ASD	Non-ASD	ASD	Non-ASD
		(*n* = 135)	(*n* = 181)	(*n* = 133)	(*n* = 183)	(*n* = 130)	(*n* = 180)	(*n* = 130)	(*n* = 276)
Communication warning behavior	>30	63 (19.94)	1 (0.31)	61 (19.30)	3 (0.95)	61 (41.22)	2 (1.35)	61 (15.02)	3 (0.74)
	≤30	72 (22.79)	180 (56.96)	72 (22.79)	180 (56.96)	69 (46.62)	16 (10.81)	69 (17.00)	273 (67.24)
	*Kappa*	0.494		0.476		0.137		0.529	
	AR	76.90%		76.26%		52.03%		82.27%	

Data are presented as n (%). TD, typically developing children; ASD, autism spectrum disorder; DLD, developmental language disorder; GDD, global developmental delay; AR, agreement rate.

### Early detection of ASD based on the communication warning behavior of CNBS-R2016

**FIGURE 3:**
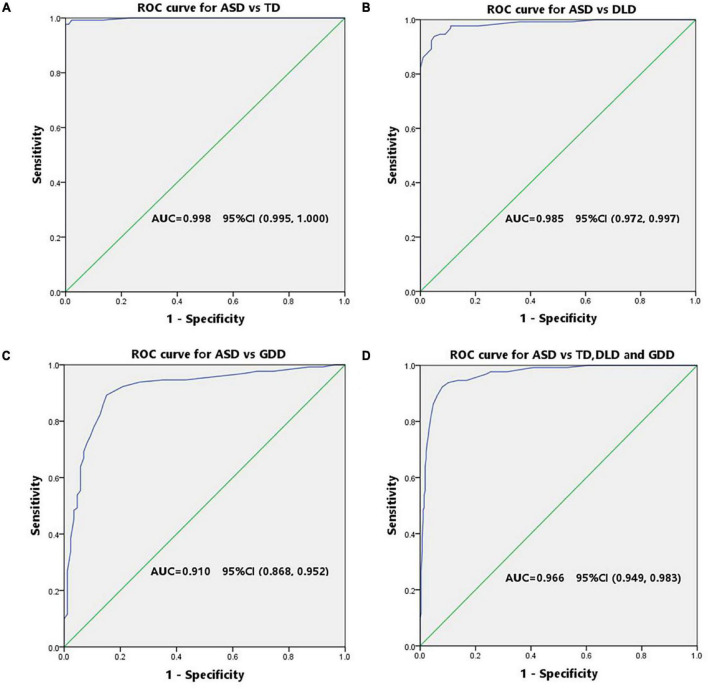
ROC curve of the communication warning behavior for screening ASD. **(A)** Receiver operator curve of ASD vs. TD. **(B)** Receiver operator curve of ASD vs. DLD. **(C)** Receiver operator curve of ASD vs. GDD. **(D)** Receiver operator curve of ASD vs. the combination of DLD and GDD.

The diagnostic validity (value for diagnostic classification) of CNBS-R2016 was analyzed by ROC analysis for ASD vs. TD, ASD vs. DLD, ASD vs. GDD, ASD vs. TD, and combination of DLD and GDD. The ROC curve plotted for the communication warning behavior scores (Figures 3A–D) determined the cutoff score on communication warning behavior that maximized sensitivity and specificity based on *Youden*’s index. Table 4 shows the corresponding AUC, sensitivity, specificity, FNR, false-positive rate, positive predictive value, and negative-predictive value for each measure. The AUC indicates the ability of the tests to correctly classify individuals with and without ASD. Excellent values of AUC were obtained in this study at each cutoff score. When we selected the communication warning behavior of CNBS-R2016 score of ≥12 points as the prediction of ASD, its consistency index with ABC, CARS, ADOS-2, and clinical diagnosis is shown in Table 5.

**TABLE 4:** Cutoff score, sensitivity, specificity, AUC, FNR, FEP, PPV, and NPV based on ROC curve analysis to discriminate ASD and TD control, as well as ASD and non-ASD clinical groups.

	Cut-off	Sensitivity	Specificity	AUC	FNR	FPR	PPV	NPV
ASD vs. TD	3.5	0.992	0.978	0.998*	0.008	0.022	0.985	0.989
ASD vs. DLD	6.5	0.977	0.890	0.985*	0.023	0.110	0.920	0.957
ASD vs. GDD	12.5	0.892	0.849	0.910*	0.108	0.167	0.906	0.841
ASD vs. TD, DLD, and GDD	12.0	0.923	0.920	0.966*	0.080	0.082	0.833	0.962

TD, typically developing children; ASD, autism spectrum disorder; DLD, developmental language disorder; GDD, global developmental delay; AUC, area under the curve; FNR, false-negative rate. *P < 0.05, statistically significant.

**TABLE 5:** Consistency between the communication warning behavior (cutoff value = 12) of CNBS-R2016 and ABC, CARS, ADOS-2, and clinical diagnosis for the classification of ASD.

		ABC		CARS		ADOS-2		Clinical diagnosis	
		ASD	Non-ASD	ASD	Non-ASD	ASD	Non-ASD	ASD	Non-ASD
		(*n* = 135)	(*n* = 181)	(*n* = 133)	(*n* = 179)	(*n* = 130)	(*n* = 18)	(*n* = 130)	(*n* = 276)
Communication warning behavior	≥12	130 (41.14)	12 (3.80)	125 (39.56)	17 (5.38)	120 (81.08)	4 (2.70)	120 (29.56)	18 (4.43)
	<12	5 (1.58)	169 (53.48)	8 (2.53)	162 (51.27)	10 (6.76)	14 (9.46)	10 (2.46)	258 (63.55)
	*Kappa*	0.891		0.816		0.613		0.844	
	AR	94.62%		90.82%		90.54%		93.10%	

TD, typically developing children; ASD, autism spectrum disorder; DLD, developmental language disorder; GDD, global developmental delay; AR, agreement rate.

## Discussion

As a native child developmental assessment tool in China, CNBS-R2016 has a unique role in cultural adaptability, and it has been widely used in child care department as a development assessment tool for routine well-child visit in Mainland China. Tang et al. had proven that the CNBS-R2016 and Griffiths Mental Development Scales (GMDS) showed good consistency in the developmental assessment of children with ASD (42). Compared with GMDS, CNBS-R2016 is more time efficient (an experienced psychologist can complete the CNBS-R2016 in 30–50 min). The communication warning behavior subscale was added to assess autism symptoms, which indicates that CNBS-R2016 not only has the potential function of screening for ASD but also has a comprehensive developmental level of children with ASD. Given the three-level child healthcare system in China, primary-level pediatricians transfer children with abnormities to higher-level medical institutions. Integrating ASD screening into routine well-child visits is helpful for the systematic monitoring of early ASD symptoms and the promotion of early diagnosis and intervention (43).

This study primarily explored whether a lower cutoff value for ASD screening is more recommended for referral in accordance with the Communication Warning Behavior of CNBS-R2016.

### Children with ASD, GDD, DLD, and TD were different from one another in the developmental assessment of CNBS-R2016

We compared the developmental level of ASD, GDD, DLD, and TD groups assessed by CNBS-R2016. With regard to the overall developmental level, the ASD group had the lowest overall DQ, followed by GDD and DLD, and they were all significantly lower than the TD group. Children in the four above mentioned groups differed from one another in the subscale of CNBS-R2016. ASD showed the lowest score in language subscale probably because speech and language problems were the main reasons that encouraged caregivers to initially seek for treatment in preschool ASD population. In addition, children in the ASD group were generally normal in gross motor but delayed in fine motor, adaptive behavior, language, and personal-social domains. The DLD group was only delayed in language, whereas the GDD group showed developmental delays in all domains. This study showed that children with different developmental disorders varied in developmental profile of CNBS-R2016. The results of CNBS-R2016 were in line with the clinical presentation of children with ASD, DLD, and GDD.

### Communication warning behavior reflected core symptoms of ASD

The communication warning behavior subscale contains 33 items. Among which, 14 items were related to social communication, 5 to restricted and repetitive behavior, 3 to language, 3 to sensory, 3 to physiological disorder in infants, 2 to intelligence, and 3 to abnormal behavior. These items served as checklists of common symptoms of ASD and referred to DSM-V (29). We revealed in our study that children with the maximum communication warning behavior scores are those with ASD. Clinical subgroups, such as GDD and DLD children, may manifest a few signs of autism symptoms, with notably higher mean communication warning behavior scores than those of the TD group. Median scores of ASD, GDD, DLD, and TD groups were 24, 8, 2, and 0, respectively, and significantly different from one another (*p* < 0.05). Li et al. (42) reported a significant positive correlation between the CNBS-R2016 communication warning behavior subscale quotient and the total ABC (*r* = 0.821, *p* < 0.001) and the total CARS (*r* = 0.734, *p* < 0.001) scores in children with ASD, respectively. ASD of preschoolers with low neurodevelopmental levels presented high scores of ABC, SRS, and CARS and a high communication warning behavior score (44). High communication warning behavior scores of children with severe autism symptoms were also verified in ASD children with sleep disorders and developmental regression (45, 46). In this study, Spearman analysis positively showed the correlations between communication warning behavior subscale quotients and scores of ABC, CARS, and ADOS-2 of the ASD group children, which were 0.812, 0.761, and 0.821, respectively (*p* < 0.05), and this result was consistent with conclusion of Li et al. (42). The communication warning behavior not only has a good correlation with ASD screening tools but also with gold standard diagnostic tool of ADOS-2. We first examined the relevance of communication warning behavior and ADOS-2 in this study and showed that communication warning behavior reflected autism core symptoms well. It could be used as an efficient ASD screening tool.

### Communication warning behavior score of 12 points is the recommended cutoff value for screening of ASD

Children with a communication warning behavior score of over than 30 points are highly suspected of ASD based on the CNBS-R2016. However, we found that some children with a communication warning behavior score of less than 30 were also diagnosed with ASD later on. In this study, children with a communication warning behavior score of below 30 points accounted for 53% of all the diagnoses of ASD. We calculated the consistency of a communication warning behavior score of over 30 points and ABC, CARS, ADOS-2, and clinical diagnosis for the prediction of ASD. Consistency indicators, including the *Kappa* value and agreement rate, were low to moderate (Table 3). Excellent specificity of 98.9% was obtained, but sensitivity of 46.9% was poor. Therefore, we aimed to explore an appropriate lower communication warning behavior score to ensure the best predictive effect of ASD. The ROC curve was used for this analysis. A cutoff point of 12 was achieved for distinguishing ASD from non-ASD, and the corresponding area under the ROC curve was 0.966, with sensitivity of 0.923 and 0.920, respectively.

The achieved cutoff point of 12 coincides with CNBS-R2016’s previous conclusion that 12–30 points indicate the potential of communication and interaction disorder. Approximately 73–80% children with moderate to severe social communication disorder were diagnosed with ASD. Other reasons include GDD or intellectual disability (GDD/ID), hearing loss, and metabolic or genetic diseases (47–49). A total of three children with a communication warning behavior score over 12 points were diagnosed with GDD but not with ASD in this study. In addition to typical abnormal behaviors, children with ASD often experience comorbidities, such as GDD/ID (25). Notably, children diagnosed with severe GDD/ID may also show autism-like symptoms (49). A rigorous diagnostic evaluation for ASD needs to be initiated to identify the diagnosis for those children. A total of ten children with a communication warning behavior score of less than 12 points were also diagnosed with ASD in this study. Further conditional analysis of these 10 children showed that three suffered from developmental regression at around 2.5 years old, without evident disorders before this age. A total of two children presented mild symptoms, and the five other ASD children were high functioning. Developmental regression is a warning behavior of ASD that requires further investigation in children with developmental regression. Although high functioning ASD children or those with mild symptoms are difficult to detect in the early stage because of the negative results of common ASD screening scales, the condition of these children is usually only recognized by experts. Therefore, it is no wonder that these seven children had lower communication warning behavior scores.

The sensitivity of 12 points was more excellent than 30, with no significant decrease in specificity. In clinical practice, the communication warning behavior is generally used to indicate children for further ASD diagnostic evaluation, thereby requiring high sensitivity. A cut off score of 12 points could serve this purpose well.

We also analyzed the best cutoff values for distinguishing ASD vs. TD, ASD vs. DLD, and ASD vs. GDD using the same method, which were 3.5, 6.5, and 12.5, respectively, and corresponding AUC value was all above 0.85, which indicated high sensitivity and specificity. Children with DLD, GDD, and ASD have a communication disorder with varying degrees. This study could be used as a reference for clinical classification among ASD, DLD, GDD, and TD.

### Recommendations

The communication warning behavior subscale of CNBS-R2016 is important for the early detection and differential diagnosis of ASD. In this study, the communication warning behavior score of 12 points was the best cutoff for screening ASD children. Therefore, we suggest that when the communication warning behavior scores are 12 points or greater, considerable attention is needed, and further comprehensive diagnostic evaluation of ASD is required in institutions that are qualified to diagnose ASD. Moreover, in primary care hospital or institutions that are not qualified to diagnose ASD, children with a communication warning behavior score of 12 points or greater should be referred to qualified institutions for diagnosis.

## Conclusion

Our study sublimated the original explanation about communication warning behavior of CNBS-R2016 and provides specific and feasible recommendations for children with communication warning behavior scores over than 12 points. A recommended cutoff point of 12 for further comprehensive diagnostic evaluation for ASD can better assist the early detection and diagnosis of ASD in China.

## Limitations and further directions

The limitations of this study must be noted to provide a comprehensive understanding of the results. First, the sample children were only recruited from Wuhan, China, and this study had a single-center design. Second, the size of the control group was smaller than that of the ASD group, and different groups were not matched in numbers. In addition, the control samples except for the TD group primarily recruited those with language disorders, and most ASD children had intellectual disabilities, which may limit the power of this study. Therefore, muti-centered and larger population with more different kinds of developmental disorders is necessary to verify the reliability of the results.

## Data availability statement

The raw data supporting the conclusions of this article will be made available by the authors, without undue reservation.

## Ethics statement

The studies involving human participants were reviewed and approved by Ethical Committee of Tongji Hospital Affiliated Tongji Medical College, Huazhong University of Science and Technology. Written informed consent to participate in this study was provided by the participants’ legal guardian/next of kin.

## Author contributions

SC, XH, JZ, and YH designed the study. SC, JL, DW, TY, LT, LX, MC, SH, and YH recruited subjects and collected medical information. SC carried out the analysis and wrote the manuscript. JZ instructed the statistical method and writing idea. XH and LT revised the manuscript. YH guided and supervised all work. All authors contributed to the article and approved the submitted version.

## Conflict of interest

The authors declare that the research was conducted in the absence of any commercial or financial relationships that could be construed as a potential conflict of interest.

## Publisher’s note

All claims expressed in this article are solely those of the authors and do not necessarily represent those of their affiliated organizations, or those of the publisher, the editors and the reviewers. Any product that may be evaluated in this article, or claim that may be made by its manufacturer, is not guaranteed or endorsed by the publisher.
